# Management of Post-Viral Postural Orthostatic Tachycardia Syndrome With Craniosacral Therapy

**DOI:** 10.7759/cureus.35009

**Published:** 2023-02-15

**Authors:** Leonid Tafler, Aysham Chaudry, Heejin Cho, Angeles Garcia

**Affiliations:** 1 Primary Care, Touro College of Osteopathic Medicine, New York City, USA; 2 Medical School, Touro College of Osteopathic Medicine, Middletown, USA; 3 Medical School, Touro College of Osteopathic Medicine, New York City, USA

**Keywords:** craniosacral therapy, postural orthostatic tachycardia syndrome diagnosis, neuropathic pots, pots covid-19, autonomic disturbance, cv4, osteopathic manipulative medicine, pots treatment, pots and covid vaccine, autoimmune pots

## Abstract

Postural Orthostatic Tachycardia Syndrome (POTS) is a rare disorder of the autonomic nervous system. The number of people afflicted with this dysautonomia has increased dramatically in recent years due to the long-term effects of coronavirus disease (COVID-19); however, it is largely underdiagnosed. This case report is about a patient with post-viral neuropathic POTS. Neuropathic POTS is believed to be due to the damage of small nerve fibers that regulate the constriction of the blood vessels in the limb and abdomen, which leads to interference with vasoconstriction, and therefore causes tachycardia. Current literature emphasizes a treatment that is based on lifestyle modifications, such as increasing water and salt intake, and symptomatic pharmacological treatment. In this case, the 39-year-old male ptient was treated with osteopathic manipulative treatment (OMT), specifically the compression of the fourth ventricle (CV4), which has been associated with the production of hyperparasympathetic and anti-inflammatory effects and, hence, helps overcome the small-fiber neuropathy caused by the viral illness. We found that the CV4 technique led to the successful remission of the patient’s symptoms. Therefore, we propose craniosacral therapy as a successful single management modality in patients with POTS.

## Introduction

Postural orthostatic tachycardia syndrome (POTS) is characterized by an abnormal autonomic nervous system response when a patient goes from a supine to an upright posture, and is a common condition affecting one to three million Americans [1]. In a healthy individual, the autonomic nervous system is activated in order to compensate for the downward displacement of blood [1]. The increase in sympathetic response results in peripheral vasoconstriction while the decrease in parasympathetic response increases the heart rate. Overall, this will allow the body to maintain perfusion throughout all vital organs. However, in POTS, an individual’s autonomic nervous system overcompensates for the postural change, resulting in tachycardia. The diagnosis of POTS can be made when a patient experiences an increase in heart rate by at least 30 beats per minute within 10 minutes of standing up in the absence of hypotension [2]. Patients may also experience symptoms of orthostatic intolerance such as, but not limited to, lightheadedness, palpitations, headaches, and nausea [2]. 

Various pathophysiologic mechanisms of POTS exist, but some common ones include hypovolemia, autoimmunity, and neuropathy. Reduction in intravascular volume can result in decreased venous return to the heart and lead to reflex tachycardia. Additionally, in up to half of POTS cases, small fiber neuropathy is present which leads to dysautonomia [2]. More recently, dysautonomia has been observed in patients with coronavirus disease 2019 (COVID-19), and POTS has been identified as a possible long-term manifestation of COVID-19. It has also been found that the severe acute respiratory syndrome coronavirus 2 (SARS‑CoV‑2) virus binds to the angiotensin-converting enzyme 2 (ACE2) receptor, possibly causing hyperadrenergic POTS [3]. Lastly, it has been noted that for a select number of POTS patients, an autoimmune mechanism of orthostatic symptoms has been found. Autoantibodies to ganglionic nicotinic acetylcholine receptors, alpha-1 adrenergic receptors, beta-1/2 adrenergic, and angiotensin II type 1 receptors have been found [4]. These may interfere with peripheral vasoconstriction and lead to tachycardia. 

Current management of POTS relies mostly on symptom management and lifestyle modifications. Patients are encouraged to increase fluid and salt intake, partake in aerobic exercise, and wear compression garments [2]. While there is no universal pharmacological guideline for POTS, medications such as fludrocortisone, pyridostigmine, midodrine, and beta blockers can help with its management, depending on the cause [2]. Beta blockers can blunt elevations in heart rate. Midodrine, an alpha agonist, constricts arterial and venous blood vessels in patients with insufficient adrenergic vasoconstriction [2]. Pyridostigmine can also enhance sympathetic response [2]. Fludrocortisone can promote renal sodium reabsorption and therefore increase intravascular volume [2]. 

Here, we present a case of POTS that was diagnosed one year after the patient was diagnosed with COVID-19. Due to the nature of POTS being an autonomic imbalance due to a small-fiber neuropathy, the patient was offered a traditional osteopathic approach and craniosacral therapy, which is postulated to restore autonomic function and balance. The patient’s symptoms improved even after one round of treatment and continued to progress positively with weekly treatments. 

Craniosacral therapy is an osteopathic technique that utilizes light touch to evaluate and balance restrictions in the craniosacral system. The flow of CSF can be measured through gentle palpation of no more than 5 grams to the patient’s head. An abnormal cranial rhythmic impulse rate can then be treated using compression of the fourth ventricle (CV4) to restore to an optimal rate. The optimal rate of the cranial rhythmic impulse is approximately 8-12 cycles per minute.

CV4 is a well-known osteopathic technique that balances sympathetic and parasympathetic branches of the nervous system. The fourth ventricle lies anterior to the occipital squama, and by applying pressure to the occiput, the physician can restore the cranial rhythmic impulse and help relieve congestion in the ventricles by encouraging the flow of CSF [5]. The CV4 technique is done with the patient lying supine and the physician's thenar eminences cradling the occiput. The physician then encourages extension while inhibiting flexion. These movements are continued until a motionless state is reached and a softening of the surrounding area occurs [6]. Contraindications to craniosacral therapy include increased intracranial pressure, intracranial hemorrhage, brain tumors, and brain aneurysms [7].

## Case presentation

A 39-year-old male with no significant past medical history presented with a 12-month history of sinus tachycardia and chest palpitations, which the patient reported started after infection with COVID-19. The patient’s medical, surgical, family, and social history was noncontributory. Physical exam at the office revealed no abnormal findings. The patient reported a resting heart rate of 125 beats per minute (bpm) and up to 150 bpm with activities, such as walking. The patient reported no relieving factors or precipitating factors. His symptoms impacted his daily activities and employment in construction, requiring him to take a leave of absence. Despite numerous visits with cardiologists, a normal complete blood count and comprehensive metabolic panel, and a negative hematology panel, his history of a previous viral illness was not taken into consideration. In reviewing his radiological history, a brain MRI was unremarkable. A bilateral lower extremity arterial doppler was normal and showed no evidence of stenosis or occlusion. In reviewing other diagnostic studies, an ECG was normal. 

A carotid doppler was performed that showed no evidence of carotid stenosis. The cardiac stress test revealed normal functional capacity for his age and non-ischemic ECG response to exercise. A baseline ECG was performed prior to the stress test and showed a normal ECG with sinus rhythm. An echocardiogram revealed structurally normal mitral and tricuspid valves, ejection fraction of 65%, and normal atrial and ventricle size. The patient was provided a 14-day Holter monitor, which detected three patient-triggered events, one normal sinus rhythm, and two sinus tachycardia events.

After his initial osteopathic assessment, the patient elected for craniosacral therapy for symptomatic treatment. After the initial application of craniosacral therapy, the patient reported improvement in symptoms and heart rate the following day, with a heart rate of 95 bpm with activity. This brief improvement was followed by a return to the previous state in the coming days, with a heart rate of up to 145 bpm with mild activity, such as walking. 

**Figure 1:**
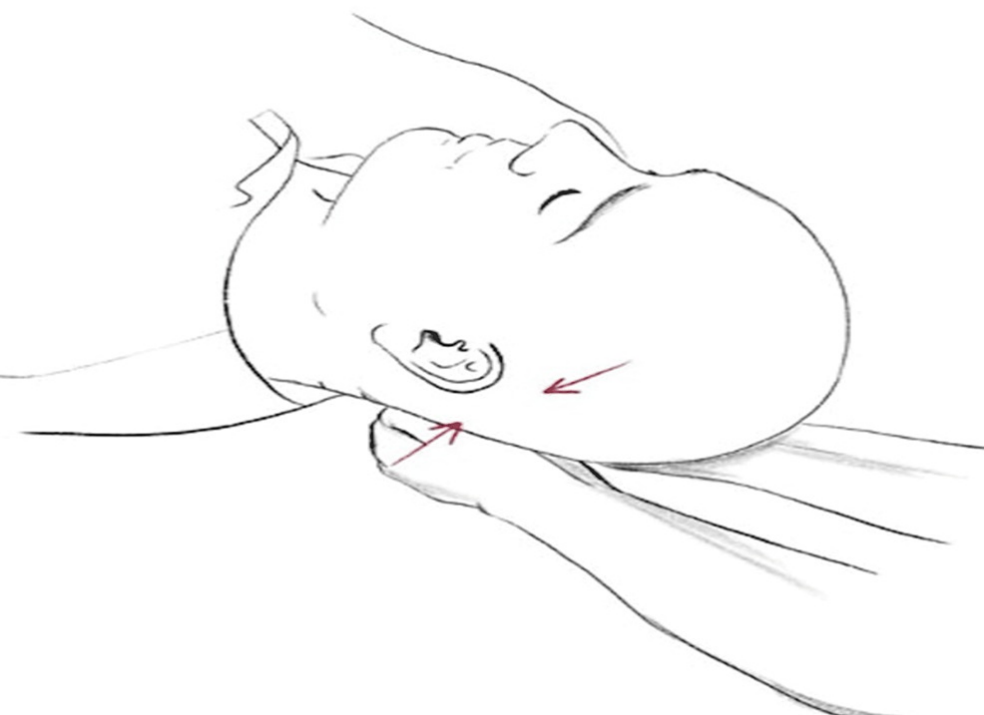
CV4 oblique view. CV4: compression of the fourth ventricle Image Source: Young et al., 2021 [7]

At a subsequent visit one week later, an active standing test performed at the office showed a 39 bpm increase in heart rate from supine to standing, with the supine heart rate at 82 bpm and the standing heart rate at 121 bpm within 10 minutes. The blood pressure during the test remained normal, with standing blood pressure at 130/80, ruling out orthostatic hypotension. Osteopathic manipulation therapy was performed using the traditional osteopathic approach and craniosacral therapy, specifically the CV4 technique (Figure 1). Re-evaluation with an active standing test showed a 12 bpm increase in heart rate from supine to standing, with the supine heart rate at 77 bpm and standing heart rate at 89 bpm within 10 minutes. 

In an additional session, the patient had a supine heart rate of 79 bpm, which increased by 30 bpm to 109 bpm within 10 minutes of standing. After the osteopathic manipulative treatment (OMT) regimen, the supine heart rate was 70 bpm and the standing heart rate was 83 bpm. 

The patient was scheduled for five additional weekly sessions of this regimen for the treatment of postural orthostatic tachycardia syndrome. On the first visit, the patient had a pre-OMT difference in heart rate from supine to standing position of 39 bpm, on the second visit there was a difference of 30 bpm, and at each subsequent visit after that, there was a change of less than 10-15 bpm (Table 1). After the second session, the patient also reported a lower heart rate throughout the week, up to 88 bpm with mild activity, such as walking. After the last session, the patient experienced relief from his symptoms of chest palpitations and tachycardia for more than six months.

**Table 1:** The change in patient’s heart rate from a supine to a standing position before craniosacral therapy. OMT: osteopathic manipulative treatment; HR: heart rate

Visit	Pre-OMT change in HR from supine to standing
First visit	39 bpm
Second visit	30 bpm
Subsequent visits	10-15 bpm

## Discussion

**Figure 2:**
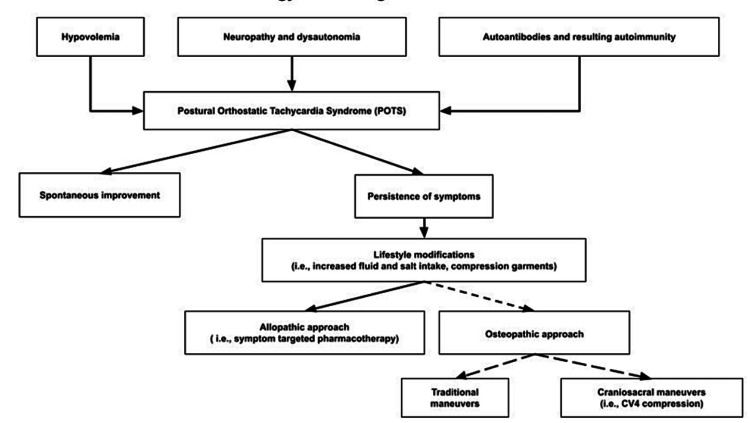
Etiology and management of POTS. POTS: postural orthostatic tachycardia syndrome

Postural orthostatic tachycardia syndrome (POTS) is a dysautonomia that has been increasing in prevalence in the United States. It is an abnormal symptomatic autonomic response to an upright position [8]. There are several proposed mechanisms of etiology for the development of the syndrome (Figure 2). The different types of POTS include neuropathic, hyperadrenergic, hypovolemic, and secondary causes [1]. 

Neuropathic POTS is associated with impaired adrenergic nerve function or damage to the small nerve fibers that regulate blood vessels in the extremities [9]. A hyperadrenergic state associated with elevated levels of norepinephrine may also lead to the syndrome. This may also present with elevated blood pressure during the Valsalva maneuver. A decreased intravascular volume can lead to hypovolemic POTS due to the required increase in cardiac output to compensate for hypovolemia [10]. Autoimmunity with crossreactivity of antibodies against various viral illnesses towards ganglionic nicotinic acetylcholine receptors, alpha-1 and beta-1 and 2 adrenergic receptors, and angiotensin type II receptors can lead to POTS [9]. Lastly, secondary causes that lead to autonomic neuropathy may be considered, such as diabetic neuropathy, Lyme disease, systemic lupus erythematosus, or Sjogren's syndrome [1]. We suspect a neuropathic cause of POTS in our patient that may be secondary to a viral cause, due to his history of infection with COVID-19. 

**Figure 3:**
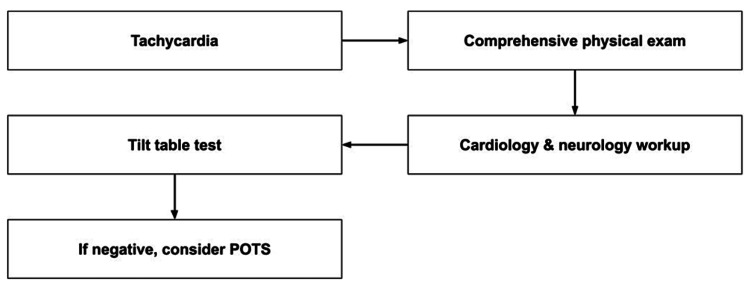
Algorithm for the diagnosis of POTS. POTS: postural orthostatic tachycardia syndrome

Despite multiple consultations with cardiology, the patient’s illness went undiagnosed. In Figure 3, we propose an algorithm for the approach to the diagnosis of POTS. A comprehensive physical exam along with CBC and CMP should be performed for unexplained sinus tachycardia. If findings are negative, a complete cardiological and neurological evaluation should be performed to rule out other causes of tachycardia, such as viral myocarditis and reflex syncope. Lastly, a tilt table test should be performed to rule out orthostatic hypotension before making the diagnosis of POTS. 

Initial management strategies include an increase in salt and fluid intake along with exercise to avoid physical deconditioning (Figure 2) [11]. Behavioral changes to reduce triggers and maintenance of an upright posture to avoid deconditioning are recommended [12]. Compression garments to reduce venous pooling may be beneficial [13]. Beta blockers can be used in patients without comorbidities [14]. Vasopressors can be used for patients with neurogenic symptoms [9]. Norepinephrine uptake inhibitors should be avoided in patients with hyperadrenergic POTS.

Due to our patient’s debilitating condition and hindered ability to partake in daily activities and employment, we devised a treatment strategy for his refractory case of POTS. We offered traditional osteopathic therapy and craniosacral therapy. The patient did not have much improvement with the traditional approach. We found craniosacral therapy, specifically the CV4 technique, to be the most effective. We utilized the CV4 technique, which is one of the widely used craniosacral osteopathic manipulations aimed at influencing the function of the nervous system, in this case, promoting a balance between a parasympathetic and sympathetic response post treatment [15]. It is believed that with manual manipulation of this system, sensory, motor, cognitive, and emotional processes in the nervous system are affected [16]. The procedure of CV4 is as follows: the patient lays down supine, the clinician holds the squamous part of the occipital bone and places gentle upward and medial pressure on the occiput, and then exaggerates the pressure during the extension phase of the occiput. This pressure is then held steady until a softening is felt, indicating release [13]. It is believed that this restores the balance of the autonomic nervous system of patients who receive this treatment. 

Statistically, POTS affects one to three million Americans [1]. The treatment approach we devised was also successfully used on another patient with POTS at our office. Despite limited data, the success seen in these two cases of a rare disease is a testament to the effectiveness of this potential treatment approach. Based on these cases, we propose that craniosacral therapy performed by a trained osteopathic physician could be incorporated into the treatment algorithm alongside traditional therapy for patients with POTS.

## Conclusions

POTS is a rare dysautonomia that has been increasing in prevalence in the United States, and one of the causes of POTS may be post-viral neuropathy. Current management of POTS is limited and is mainly based on lifestyle modifications and medications. We believe craniosacral technique, namely CV4, will be beneficial for patients with POTS when performed as a main therapy or in adjunct to traditional therapy. This technique can be successfully used to reduce the frequency of flares in a chronic condition for long-term management.
